# Efficacy of Several Types of Pest Bird Deterrents and General Trend of Pest Birds at an Industrial Factory

**DOI:** 10.21315/tlsr2025.36.1.7

**Published:** 2025-03-30

**Authors:** Imran Mohd Hornain, Nik Fadzly N Rosely

**Affiliations:** School of Biological Sciences, Universiti Sains Malaysia, 11800 USM Pulau Pinang, Malaysia

**Keywords:** Barn Swallow, *Hirundo rustica*, Bird Deterrent Methods, Roosting, Industrial Plant, Migration, Burung Suolo Api, *Hirundo rustica*, Kaedah Pencegahan Burung, Bertenggek, Kilang Perindustrian, Penghijrahan

## Abstract

Controlling pest birds is a complex problem, especially for large areas, compared to individual homes. This study presents a new perspective on pest birds plaguing a large semiconductor factory. We evaluated the efficacy of nine bird deterrent methods: alarm and distress calls broadcasted from portable speaker, sound frequencies ranged from (i) 24.5 kHz–45.5 kHz; and (ii) 13.5 kHz–45.5 kHz together with flashing lights emitted from sonic device, methyl anthranilate (MA), moving and static bird predator models, reflective compact discs, high and low visibility reflective tapes in deterring Barn Swallow (*Hirundo rustica*), Pacific Swallow (*Hirundo tahitica*) and Asian Glossy Starling (*Aplonis panayensis*) from perching on targeted spots. Monthly counts of pest birds roosting at study site were also conducted. Our results showed that alarm and distress calls broadcasted from portable speaker, sound frequencies ranged from 13.5 kHz–45.5 kHz together with flashing lights emitted from sonic device, moving and static bird predator model, reflective compact discs, high and low visibility reflective tapes were significantly effective in deterring pest birds from targeted spots. The pest bird population reached its peak (35,063) in January 2021, while the least (3,685) was recorded in May 2021. The effectiveness of pest bird deterrents might be influenced by the quantity and method of deployment.

## Abstract (fr)

**Abstrak:**

Mengawal burung perosak adalah masalah yang kompleks, terutamanya kawasan yang luas, berbanding dengan rumah individu. Kajian ini membentangkan perspektif baharu tentang burung perosak yang menyerang sebuah kilang semikonduktor. Kami menilai keberkesanan sembilan kaedah pencegahan burung: panggilan penggera dan kecemasan yang disiarkan daripada pembesar suara mudah alih, frekuensi bunyi berjulat daripada (i) 24.5 kHz–45.5 kHz; dan (ii) 13.5 kHz–45.5 kHz bersama-sama lampu berkelip yang dipancarkan daripada peranti sonik, metil anthranilate (MA), model pemangsa burung bergerak dan statik, cakera padat reflektif, pita reflektif keterlihatan tinggi dan rendah untuk menghalang Burung Suolo Api (*Hirundo rustica*), Burung Layang-layang Batu (*Hirundo tahitica*) dan Burung Perling Mata Merah (*Aplonis panayensis*) daripada hinggap di tempat yang disasarkan. Kiraan bulanan burung perosak yang bertenggek di tapak kajian turut dijalankan. Keputusan kami menunjukkan bahawa panggilan penggera dan kecemasan daripada pembesar suara mudah alih, frekuensi bunyi berjulat dari 13.5 kHz–45.5 kHz bersama-sama dengan lampu berkelip yang dipancarkan daripada peranti sonik, model pemangsa burung bergerak dan statik, cakera padat reflektif, pita reflektif keterlihatan tinggi dan rendah menjadi ketara berkesan dalam menghalang burung perosak dari tempat yang disasarkan. Populasi burung perosak mencapai kemuncaknya (35,063) pada Januari 2021 manakala sekurang-kurangnya 3,685 dicatatkan pada Mei 2021. Keberkesanan penghalang burung perosak mungkin dipengaruhi oleh kuantiti dan kaedah penyebaran.

**Highlights**

- **Effectiveness of Deterrents:** Our results showed that alarm and distress calls broadcasted from portable speakers, sound frequencies ranging from 13.5 kHz–45.5 kHz together with flashing lights emitted from sonic devices, moving and static bird predator models, reflective compact discs, high and low visibility reflective tapes were significantly effective in deterring pest birds from targeted spots. The most effective were the moving predator model, reflective compact disc and high visibility reflective tapes.
- **Population Trends:** The pest bird population peaked in January 2021 (35,063 individuals) and reached its lowest in May 2021 (3,685 individuals), influenced by migratory patterns and mitigation measures.
- **Deployment Considerations:** The success of deterrent methods depends on deployment strategies, including quantity, placement and combination of multiple techniques to prevent habituation and enhance efficacy.

## INTRODUCTION

Due to the loss of natural habitats such as forests, grasslands and wetlands, many birds rely on crops, aquaculture farms and man-made structures. Subsequently, these groups of birds are labelled as pest animals since they are causing multiple problems such as crop depredation, noise, bird droppings, aesthetic and health issues (Anderson *et al*. 2013; Haag-Wackernagel & Geigenfeind 2008; Sausse & Lévy 2021). The most common pest birds are Feral Pigeon (*Columba livia*), House Sparrow (*Passer domesticus*), Asian Glossy Starling (*Aplonis panayensis*), Common Myna (*Acridotheres tristis*) and House Crow (*Corvus splendens* L.) in Malaysia as well as in many countries around the world (Arazmi *et al*. 2022; Shieh *et al*. 2016; Johan *et al*. 2022). Pest birds can cause aggregate damage to blueberry, wine grape, honeycrisp apple, sweet and tart cherry crops in five different states in United States up to USD189 million, and the cost of managing bird damage was estimated to be at USD737 million (Anderson *et al*. 2013).

A few bird deterrent methods have been used globally to manage pest birds, generally categorised as visual deterrents, auditory deterrents, chemical deterrents, natural predation, habitat modification, physical exclusion and lethal techniques (Bishop *et al*. 2003). Many visual deterrents use a perceived threat or a visual disturbance to scare the birds away. Examples of visual deterrents are reflective tapes, scarecrows, mirrors and reflectors, bird predator models, balloons with eyespots, kites and flags (Cantlay *et al*. 2020; Wang *et al*. 2020). Birds may habituate to these methods if they are exposed for some time. Auditory deterrents such as bioacoustics and anthropogenic sound produced by sonic devices, gas cannons and firecrackers are practical too; however, just like visual deterrents, birds may habituate to them quickly, thus reducing their efficacy over time (Pruteanu *et al*. 2023). Plus, using auditory deterrents can raise noise issues, so it is impractical to use them near human residential areas. Bioacoustics such as alarm calls are generally used to warn other birds about the threat’s presence or signal to the predator that it has been detected, while distress calls are produced when captured by a predator (Griffin 2008). A few studies have been conducted to evaluate the efficacy of methyl anthranilate (MA) in deterring blackbird from rice and sunflower fields (Werner *et al*. 2005); dispersed large number of swallows and killdeer at the airport to prevent bird strike (Engeman *et al*. 2002); and deter red-winged blackbirds by using MA as an avian feeding deterrent (Avery *et al*. 1995).

Research on bird deterrent methods has focused mainly on protecting crops, plantation trees, fish farms and preventing aircraft strikes at the airport. However, less information is available to deter pest birds from man-made structures such as industrial areas. One of the semiconductor factories in Kulim Hi-Tech Park, Kedah, Malaysia is being plagued by thousands of birds, specifically Barn Swallow (*Hirundo rustica*), Pacific Swallow (*Hirundo tahitica*) and Asian Glossy Starling (*Aplonis panayensis*) (Appendix A). These pest birds appear in semiconductor factory airspace from 1830 until 1930 and eventually land on factory structures for roosting (Appendix B). Consequently, their droppings have raised health issues and caused damage to factory structures.

The first objective of this study is to investigate the efficacy of the following methods to deter pest birds from roosting on industrial structures: alarm and distress calls broadcasted from portable speakers, sound frequencies ranged from (i) 24.5 kHz–45.5 kHz; and (ii) 13.5 kHz–45.5 kHz together with flashing lights emitted from sonic device, MA, moving and static bird predator models, reflective compact discs, high and low visibility reflective tapes. The frequencies were already programmed into the device. Pruteanu *et al*. (2023) reviewed the numerous frequencies that are commonly used in scaring birds and the preprogrammed sound range fits into frequencies commonly known to scare off birds. The second objective is to investigate the general trend of pest bird population in semiconductor factory areas for one year.

## MATERIALS AND METHODS

### Study Site

The study was conducted at one of the semiconductor factories in Kulim Hi-Tech Park at the coordinate of 5°23′57″ N 100°35′34″ E (Appendices C and D). Kulim Hi-Tech Park is an industrial park dedicated to high technology enterprises in Kulim District, Kedah, Peninsular Malaysia. The semiconductor factory is surrounded by forest and human residential areas. However, one of the forest areas has turned into empty land as the company expanded its production capacity. The total area of the semiconductor factory is about 0.25 km^2^ which is mainly comprised of its car park, office buildings (Office 1 and Office 2), fabrication buildings (FAB 1 and FAB 2), centre utility buildings (CUB 1 and CUB 2) and water tank area. Based on previous observations, there are two bird hotspot areas; the first is between the Office 1 and FAB 1 building, while the other is between the FAB 1 and FAB 2 building.

All bird deterrent methods were set up randomly on top of the selected study spots (study spot A or study spot B) located at the FAB 2 building (Appendix E). These factory structures were selected because the height of these structures enables us to conduct the experiment efficiently compared to other factory structures located about 4 m above the ground, which required a scaffold or crane to reach.

### Experimental Design and Data Collection

#### Efficacy of pest bird deterrents

All bird deterrent methods were tested for 60 min only as we wanted to see whether these pest birds could habituate to our deployed treatments in a short period before implementing them for future use. For each 10 min, a snapshot of the number of birds perched on top of selected study spots (study spot A or study spot B) was taken by using a Nikon Coolpix P900 camera with a 24–2,000 mm lens. The observer took the snapshots from another spot on the middle rooftop located at the opposite building, FAB 1 building. We also took a snapshot of a number of birds perched before treatments were set up. All treatments were deployed ranged from 2000 h–2200 h with only one or two treatments per day due to the limited availability of study spots. Since this was a free-ranging study, our treatment might be exposed to external variables such as heavy vehicles and factory personnel. To counter this, we also collected data from when no deterrent was introduced at study spots as a control treatment.

There are nine types of bird deterrent methods used in this study: alarm and distress calls broadcasted from portable speaker, sound frequencies ranged from (i) 24.5 kHz–45.5 kHz; and (ii) 13.5 kHz–45.5 kHz together with flashing lights emitted from sonic device, MA, moving and static bird predator model, reflective compact disc, high and low visibility reflective tapes (Appendix F).

The portable speaker and sonic device were placed in the middle of the study spots. A portable speaker broadcasted alarm and distress calls made by Barn Swallow (*Hirundo rustica*) and a variety of bird predator recordings made by Eurasian Sparrowhawk (*Accipiter nisus*), Peregrine Falcon (*Falco peregrinus*), Chinese Sparrowhawk (*Accipiter soloensis*), and Japanese Sparrowhawk (*Accipiter gularis*). These bird sound recordings were taken from the Xeno Canto website (www.xeno-canto.org) and were played randomly in sequence and continuously within 60 min. Our sonic device can emit two ranges of sound frequencies: (i) 24.5 kHz–45.5 kHz and (ii) 13.5 kHz–45.5 kHz with flashing lights. In addition, both can also emit audible alarms and were switched on during this study. We used MA that was formulated as Bird-X Bird Stop. About 10 mL of MA was poured into our portable mist machine and was mixed with 490 mL of tap water to make 2% of the solution. The portable mist machine was put in the middle position on top of the study spots and continuously produced mist for 60 min.

The moving and static bird predator model was hung using a metal hook under the upper-level awning and in the middle position of the study spots. The movable bird predator model was powered by two AA batteries that could last about four to five hours. Our moving bird predator model moved by flapping its wing and emitting sound and flashing red light through its LED eyes. Three units of the reflective compact disc were also hung under an upper-level awning using a metal hook and additional fishing line. Each reflective compact disc was separated about 2.7 m horizontally from each other. The distance between the centre of the disc to the surface of selected study spots was about 0.1 m. High- and low-visibility reflective tapes were set up on top of study spots horizontally in three separate lines 0.5 m apart. The length of these tapes were 10 m in length and 0.05 m in width. Both types of reflective tapes were placed at study spots with double-sided tape to prevent them from falling to the ground. High visibility reflective tape consists of an iridescent checkered pattern printed on it, which refracts the light-producing rainbow colours, while low visibility reflective tape consists of a honeycomb plain pattern, which generally does not refract any rainbow colours. High visibility reflective tape also has an audible element as these tapes would produce a crackling sound caused by a strong wind.

#### General trend of pest birds

It was difficult to count all three species of pest birds separately and to differentiate Barn Swallow from Pacific Swallow in far distance and dark environments; thus, all these pest bird species calculations were grouped into single calculations. Monthly counts were made about three hours in duration, usually from 2100 h–2400 h depending on the weather condition and were conducted on the fourth or fifth week of each month for 12 months, starting from January to December 2021. We conducted pest bird population counting at each part of the factory’s main buildings (Office 1 and Office 2, FAB 1 and FAB 2, CUB 1 and CUB 2) and the areas opposite CUB 1 and CUB 2. Counts were made using block-counting methods (Medway 2008), i.e., the average number of pest birds settled on linear perching spots multiplied by the total number of same perching spots occupied. Also, counts were always made by a single observer to keep consistency. For the irregular pattern of roosting sites such as under awning areas, building ledges and secluded areas that were difficult to count, counts were estimated in the multiple of 10 individuals.

### Statistical Analysis

#### Efficacy of pest bird deterrents

The data were tabulated in percentage as the initial number of pest birds perched on study spots for each trial differed. All data for repeated trials were summarised into a mean percentage. The data were analysed using Statistical Package for the Social Sciences (SPSS) version 27. We used a two-way analysis of variance (ANOVA) to determine the effect of our independent variables (bird deterrent methods and timing) on a dependent variable (mean percentage of birds). Statistical significance was set at *p* < 0.05.

## RESULTS

### Efficacy of Pest Bird Deterrents

**Figure 1:**
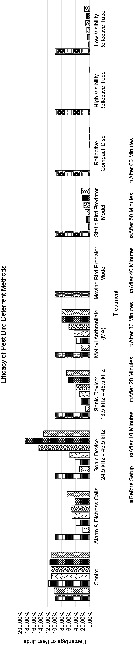
Mean percentage of pest birds for all types of bird deterrents. The control percentages are normal observations without any deterrent setup at the studied spots.

Among nine bird deterrent methods used in this study, the moving bird predator model recorded the lowest mean percentage of pest birds (0.00%) with a constant trend along 60 min duration (Fig. 1). High visibility reflective tapes and reflective compact discs recorded the second- and third-lowest mean percentage of birds (0.09% and 0.41%, respectively) at the 60th minutes with a slightly constant trend along 60 min duration. Low visibility reflective tapes recorded the fourth-lowest mean percentage of pest birds at 60th minutes (15.02%), followed by static bird predator model (24.00%), alarm and distress calls (65.32%), sonic device with sound frequency of 13.5 kHz–45.5 kHz and flashing lights (66.61%), MA (79.44%) and the highest mean percentage of pest birds at 60th minutes was recorded by sonic device with sound frequency of 24.5 kHz–45.5 kHz (131.61%). Low visibility reflective tapes, static bird predator model and sonic device with sound frequency of 13.5 kHz–45.5 kHz with flashing lights showed an increasing trend. These trend were also shown in other similar research studies (Parrott & Watola 2008; Klug *et al*. 2023). In contrast, alarm and distress calls broadcasted from portable speaker and MA showed a slightly fluctuating trend along 60 min duration. Besides, sonic device with sound frequency of 24.5 kHz–45.5 kHz fluctuates dramatically from 29.56% at the 10th minute to 184.94% at the 50th minutes, exceeding the initial mean percentage. As a result, the mean percentage of pest birds for control treatment fluctuated from 100.00% to 119.64% along 60 min duration.

A two-way ANOVA was conducted to examine the effect of bird deterrents and timing on the percentage of pest birds. The results of the two-way ANOVA showed that there was a significant main effect of bird deterrents on the percentage of birds (F(9,140) = 11.61, *p* < 0.05, η_p_^2^ = 0.427). Post hoc comparisons using Tukey HSD test indicated the mean score for the moving bird predator model (M = 14.29, SD = 35.86), static bird predator model (M = 27.10, SD = 31.81), alarm and distress calls broadcasted from portable speaker (M = 53.17, SD = 29.72), reflective compact disc (M = 14.53, SD = 35.76), high visibility reflective tape (M = 14.30, SD = 35.85), low visibility reflective tape (M = 22.29, SD = 33.55) and sonic device with sound frequencies of 13.5 kHz–45.5 kHz with flashing lights (M = 47.80, SD = 35.52) were significantly different from control treatment (M = 109.44, SD = 16.45). However, MA (M = 61.19, SD = 45.67) and sonic device with sound frequency of 24.5 kHz–45.5 kHz (M = 98.73, SD = 126.64) did not significantly different from control treatment. In addition, there was also a significant main effect of timing on the percentage of birds (F(6,140) = 9.46, *p* < 0.05, η_p_^2^ = 0.289). Post hoc comparisons using Tukey HSD test indicated the mean score for after 10 min (M = 19.52, SD = 31.89), 20 min (M = 28.15, SD = 38.72), 30 min (M = 31.56, SD = 38.32), 40 min (M = 44.00, SD = 70.93), 50 min (M = 51.64, SD = 90.71) and 60 min (M = 49.12, SD = 68.47) were significantly different from pre-treatment (M = 100.00, SD = 0.00). In contrast, there was no significant interaction between bird deterrents and timing on percentage of birds (F(54,140) = 0.80, *p* > 0.05, η_p_^2^ = 0.235). These findings suggest that different types of bird deterrents and timing may affect the percentage of birds perching on study spots independently. The lack of a significant interaction (F(54,140) = 0.80, *p* > 0.05, η_p_^2^ = 0.235) indicates that the impact of bird deterrents on the percentage of birds remains relatively consistent regardless of the timing of the observations. This suggests the different deterrent methods have a similar effect across the time intervals studied.

### General Trend of Pest Birds

**Figure 2:**
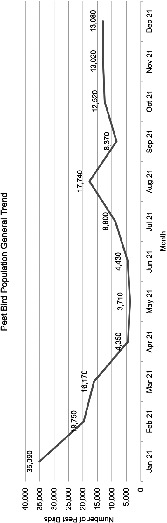
Pest bird population over 12 months period.

The peak pest bird population was recorded at 35,063 in January 2021, while the least was recorded at 3,685 in May 2021 (Fig. 2). The pest bird population maintained a similar trend between April (4,347) to June (4,424) 2021, and October (12,459) to December (13,055) 2021. An inclining trend was observed from June (4,424) to August (17,727) 2021 and September (8,328) to October (12,459) 2021, while the declining trend was observed from January (35,063) to April (4,347) 2021 and August (17,727) to September (8,328) 2021.

## DISCUSSION

### Efficacy of Pest Bird Deterrents

Our results indicated alarm and distress calls from portable speaker, sonic device with sound frequency of 13.5 kHz–45.5 kHz, moving and static bird predator model, reflective compact disc, high and low visibility reflective tapes were effective in deterring pest birds from perching at study spots within 60 min. Consequently, these pest birds would be roosting at other untreated spots within the factory compound while these deterrents were in place at study spots. On the other hand, sonic device with sound frequency of 24.5 kHz–45.5 kHz and chemical bird repellent were ineffective in deterring pest birds from perching at study spots within 60 min. Most previous studies deter pest birds from entering their foraging ground. In contrast, our objectives in this study were to deter birds from roosting on targeted spots in man-made structures. Alarm and distress calls from portable speaker was able to reduce the number of pest birds perching on top of studied spots same as other studies (Delwiche *et al*. 2005; Cho *et al*. 2020; Berge *et al*. 2007; Mahjoub *et al*. 2015). Based on Delwiche *et al*. (2005), the efficacy of bird recordings did not depend on the sound intensity but the ability of the targeted birds to recognise it.

Our sonic device model uses an infrared sensor to detect pest birds’ presence and emit an ultrasonic sound when birds are detected near the device. Based on our observation throughout the study, it seems that our sonic device model sometimes failed to detect the pest birds as we could not hear any sound or flashes emitted from our sonic device model when the pest birds were present at our study spots. Plus, sonic device with sound frequency of 24.5 kHz–45.5 kHz failed to deter pest birds as birds cannot detect ultrasonic waves due to their hearing range, similar to humans’ hearing range (Hamershock 1992). Sonic device with sound frequency of 13.5 kHz–45.5 kHz with flashing lights was effective probably due to the overlapping of their hearing range and the additional visual scaring component present.

Due to high visibility reflective tape properties, it could deter pest birds from perching onto studied spots longer than low visibility reflective tape as birds are likely to notice the visual change. Plus, with an additional auditory element, high-visibility reflective tape could also prevent birds from perching onto studied spots longer compared to low-visibility reflective tape. Previous studies investigating the efficacy of high visibility tape in deterring Mute Swans (*Cygnus olor*) from fields also found similar results (Parrott & Watola 2008). Bird predator model that appears close to lifelike through motion with startling sound can give the most significant deterrent effect (Marsh *et al*. 1992). Plus, our movable bird predator model comes with loud, startling sounds and flashing red lights through its LED eyes which enhances its efficacy. In contrast, our static bird predator model does not emit any sound and light, thus limiting its efficacy over time. Incorporating unpredictable loud sounds along with movement can enhance the effectiveness of scarecrows; however, most birds tend to habituate when these are used for longer periods (Klug *et al*. 2023).

During a windy environment, our reflective compact discs were rotated to another angle. From our observation, the pest birds would fly away when the reflective side of the disc was facing the birds but did not fly when the dull side of the disc was facing them. Furthermore, these reflective discs have a radius of deterrent effect as no bird perched near them in a circular pattern. Future studies need to be conducted to determine this radius of the deterrent effect. Our MA as chemical bird repellent did not yield effective results in this field trial. Unlike other findings, MA has been highly effective in deterring pest birds from targeted locations due to the different equipment and techniques applied where a fogging machine was used to produce a larger droplet size and cover bigger space areas with windy conditions (Engeman *et al*. 2002). However, other research findings found that MA did not prove to be effective in preventing bird damage to crops, as the use of MA has caused greater bird activity within the treatment plot, resulting in more damage to the plant. Furthermore, based on our observation, the pest birds were scattered similarly on top of the awning, which suggested that MA could not successfully deter these pest birds.

Our result showed that the steep declining trend of the pest bird population in the studied factory area from January until April 2021 was due to the migration period of Barn Swallow to the northern hemisphere region. This trend also suggested that the majority of pest bird population roosting in this factory were comprised of migrant species, Barn Swallow rather than resident species, Pacific Swallow and Asian Glossy Starling. Fazi *et al*. (2024) also found out that number of Barn Swallow at their study site in Negeri Sembilan, Peninsular Malaysia to be in similar trend which number of Barn Swallows exhibited minimal presence from January to April. During daylight, passage and wintering Barn Swallow feed widely in nearby rural areas. The swallows then congregate at nocturnal roosts in the evenings, most prominently on top of awning lining, building ledges, under awning areas, near building windows, and other factory structures, as most of these spots were illuminated with lights. Other study conducted by Mansor *et al*. (2020). We also found a similar situation where the studied Swallow population in the neighbouring towns of Bentong, Karak and Raub was roosting on utility wires illuminated with street lights. The pest bird population was also observed to congregate between tall buildings: Office 1 and FAB 1 building and along FAB 1 and FAB 2 building (Appendix D). The reason behind this is probably related to anti-predator behaviour.

### General Trend of Pest Birds

The trend of pest bird population remained lowest and similar between non-migrating period (April–June 2021) suggested that the number of Pacific Swallow and Asian Glossy Starling were around 3,500–4,500 individuals. The pest bird population increased steeply in August 2021 probably due to the emergence of young chicks of Asian Glossy Starling as reproductive season of this species in Peninsular Malaysia occurred from January to August (Shieh *et al*. 2016). Plus, we also noticed an increasing number of swallow species at other factory structures located in open space areas, which are usually undisturbed. This is probably due to some new individuals from their first migratory journey attempting to adapt to their new roosting site. However, the pest bird population trend declined steeply in September 2021 due to the large scale of mitigation measures deployed by factory personnel which include the deployment of electronic firecrackers, high visibility reflective tapes, netting, bird spikes, lasers, nest removal and industrial grade of bird chemical dispenser. The pest bird population then reached a stabilised threshold from October to December 2021 probably due to habituation. Our study is the first to investigate the trend of pest bird population, mainly swallows, in industrial areas.

## CONCLUSION

Instead of ultrasonic frequencies, wildlife managers can use infrasound frequencies (< 20 kHZ) to deter pest birds since most birds are sensitive to these hearing frequencies (Mahjoub *et al*. 2015). Besides, using an advanced technology sensor that can detect the birds in an extreme outdoor environment and with the proper placement of the acoustic product can help to improve the accuracy in detecting pest birds, thus providing effective results in deterring pest birds from targeted areas. On the other hand, using auditory deterrents such as ultrasonic devices, loudspeakers, etc., can cause noise pollution in surrounding areas. Therefore, we recommend using these deterrents far from human residential and working areas.

Chemical repellent is best used in the form of an aerosol. During windy conditions, using the right equipment can disperse pest birds in huge areas. Effigies such as the bird predator model, especially movable ones, require power input. We recommend using the bird predator model that uses DC as its energy source to ease the burden of changing batteries. Using compact discs with reflective elements on both sides of its surface produces more excellent deterrent effects towards pest birds. As for reflective tapes, instead of sticking permanently, we suggest hanging these reflective deterrents as they can produce crackling sound, thus providing more excellent protection to targeted spots from pest birds. Plus, these reflective tapes need to be replaced once in 2 to 3 weeks to maintain efficacy.

This study indicated that alarm and distress calls emitted from portable speaker, sonic device with sound frequency of 13.5 kHz–45.5 kHz with flashing lights, moving and static bird predator model, reflective compact disc, and high and low visibility reflective tape effectively deter targeted pest birds from perching onto targeted spots within 60 min independently. However, as stated by other research findings, any bird deterrent method was best to be deployed in multiple types at one time and in random patterns to slow down the habituation rates as habituation is inevitable. Plus, frequently changing the location of these deterrents would prolong the efficacy rates. The efficacy of any bird deterrent to deter pest birds from the intended area depends on the quantity of the unit deployed, deployment method, reaction of targeted species and size of targeted areas. Our experiments were not affecting the whole population of pest birds at the studied factory, but only a small population. Thus, future study needs to be conducted to investigate the efficacy of bird deterrents on a large scale.

## APPENDICES

### Appendix A

From left to right; Barn Swallow (*Hirundo rustica*), Pacific Swallow (*Hirundo tahitica*) and Asian Glossy Starling (*Aplonis panayensis*).

### Appendix B

From top left to top right; under awning surface, on top of awning surface, on cable tray. From bottom left to bottom right; along electrical conduit, on angle support, along pipelines.

### Appendix C

Location of study site (*Source*: Google Earth).

### Appendix D

Red squares indicate bird hotspot areas (*Source*: Google Earth).

### Appendix E

Left; study spot A. Right; study spot B. Both study spots are 10.75 m in length and 1.70 m in width. Red lines indicated areas where pest birds would be counted during trial.

### Appendix F

From the first row left to right; portable speaker, sonic device, methyl anthranilate with portable mist machine, moving bird predator model. From the second row left to right; reflective compact disc, high visibility reflective tape, low visibility reflective tape and static bird predator model.
